# Algorithmic Design of 3D Wireframe RNA Polyhedra

**DOI:** 10.1021/acsnano.2c06035

**Published:** 2022-09-30

**Authors:** Antti Elonen, Ashwin Karthick Natarajan, Ibuki Kawamata, Lukas Oesinghaus, Abdulmelik Mohammed, Jani Seitsonen, Yuki Suzuki, Friedrich C. Simmel, Anton Kuzyk, Pekka Orponen

**Affiliations:** † Department of Computer Science, Aalto University, 00076 Aalto, Finland; ‡ Department of Neuroscience and Biomedical Engineering, Aalto University, 00076 Aalto, Finland; § Department of Robotics, Graduate School of Engineering, Tohoku University, Sendai 980-8577, Japan; ∥ Natural Science Division, Faculty of Core Research, Ochanomizu University, Tokyo 112-8610, Japan; ⊥ Physics Department E14, Technical University Munich, 85748 Garching, Germany; # Department of Biomedical Engineering, San José State University, San José, California 95192, United States; ∇ Department of Applied Physics and Nanomicroscopy Center, Aalto University, 00076 Aalto, Finland; ◆ Frontier Research Institute for Interdisciplinary Sciences, Tohoku University, Sendai 980-8577, Japan; ¶ Division of Chemistry for Materials, Graduate School of Engineering, Mie University, Tsu 514-8507, Japan

**Keywords:** RNA origami, wireframe, polyhedra, kissing loops, self-assembly, cryo-EM

## Abstract

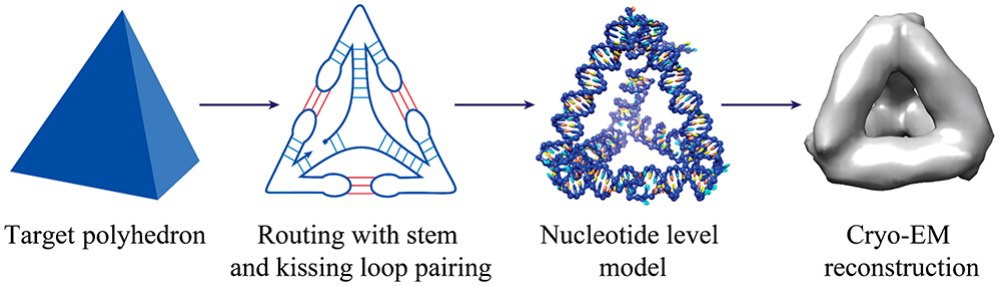

We address the problem of de novo design and synthesis of nucleic acid nanostructures, a challenge that has been considered in the area of DNA nanotechnology since the 1980s and more recently in the area of RNA nanotechnology. Toward this goal, we introduce a general algorithmic design process and software pipeline for rendering 3D wireframe polyhedral nanostructures in single-stranded RNA. To initiate the pipeline, the user creates a model of the desired polyhedron using standard 3D graphic design software. As its output, the pipeline produces an RNA nucleotide sequence whose corresponding RNA primary structure can be transcribed from a DNA template and folded in the laboratory. As case examples, we design and characterize experimentally three 3D RNA nanostructures: a tetrahedron, a triangular bipyramid, and a triangular prism. The design software is openly available and also provides an export of the targeted 3D structure into the *oxDNA* molecular dynamics simulator for easy simulation and visualization.

Nucleic acid nanotechnology, often more narrowly called *DNA nanotechnology*, uses nucleic acids as fabrication material for self-assembling nanoscale structures and devices.^1^ Major advances in this area, specifically in the self-assembly of structures, include the early multistranded DNA cube and truncated octahedron designs by Seeman et al.,^2,3^ the mostly single-stranded DNA octahedron by Shih et al.,^4^ and the fundamental DNA origami technique by Rothemund^5^ with its further applications to highly complex 2D^6−8^ and 3D^6,7,9−13^ designs.

While most current research in nucleic acid nanotechnology focuses on DNA-based nanostructures, there is also an emerging research tradition of using RNA as the fundamental substrate. One appeal of this alternative is that, while the production of designed DNA nanostructures typically proceeds by a multistage laboratory protocol that involves synthesizing the requisite nucleic acid strands and hybridizing them together in a thermally controlled process, RNA nanostructures can, in principle, be produced in quantity by the natural process of polymerase transcription from a representative DNA template, isothermally at room temperature. The challenge, however, is that the self-assembly by folding of single-stranded RNA designs is not yet as robust and predictable as the self-assembly by hybridization of scaffold and staple DNA strands, following Rothemund’s origami technique.^5^

The primary design methodology in this area of *RNA nanotechnology*, pioneered by Westhof, Guo, Jaeger et al.^14,15^ has been *RNA tectonics,*(16−18) in which naturally occurring RNA motifs are assembled by connector motifs such as kissing hairpin loops^17,19^ or single-stranded sticky ends^20^ into larger complexes. In a landmark article, Geary et al.^21^ introduced the complementary approach of *RNA origami*, wherein a single long RNA strand is folded directly into a structure that conforms to a prescribed design. In this approach, kissing hairpin loops have a central role as connectors used to bring regions of the target structure together, but except for this use of the kissing loop motif, the method is *de novo*. Besides these two general approaches, one may mention also the work of Afonin et al.,^22^ where RNA cubes are constructed from a small number of intertwined short RNA strands, and that of Han et al.,^23^ where 2D RNA tiles of various shapes are created by locking antiparallel overlays of partially complemented regions together with cohesive parallel crossover connections.

The seminal article by Geary et al.,^21^ together with its companion paper^24^ on the detailed design principles, focused on the task of producing 2D “RNA origami tiles”, somewhat analogous to the DNA origami tiles introduced by Rothemund.^5^ The basic methodology, however, carries within it a potential for similar extensions as those that followed the introduction of DNA origami by Rothemund.^5^ A major step in this direction was taken by Li et al.,^25^ who presented designs and experimental characterizations of several 2D structures and a 3D tetrahedron. The versatility of the methodology was advanced by Liu et al.,^26^ who introduced, among other things, a “branching” kissing-loop connector motif that can be used to create trivalent branches in the structures, leading to a richer design space than was previously available. A further improvement was presented by Geary et al.^27^ by enabling cotranscriptional folding of RNA origami of larger constructs compared to their earlier work.^21^

In the present work, we contribute a solution to a broad family of further design challenges in RNA nanotechnology by providing a fully general design scheme and automated software pipeline for designing *arbitrary 3D RNA wireframe polyhedra*, in fact, even arbitrary straight-line 3D RNA meshes. Analogous schemes have been presented in recent years for 3D DNA wireframe structures,^12,13,28^ but because of the differences in the substrate, these multihelix DNA origami-based techniques do not carry over to RNA. To demonstrate our method, we display designs and experimental characterizations of three simple 3D wireframe structures: a tetrahedron (similar to Li et al.),^25^ a triangular bipyramid and a triangular prism. (For the sake of brevity, we henceforth refer to the triangular bipyramid and triangular prism simply as bipyramid and prism, respectively.)

## Results and Discussion

**Figure 1:**
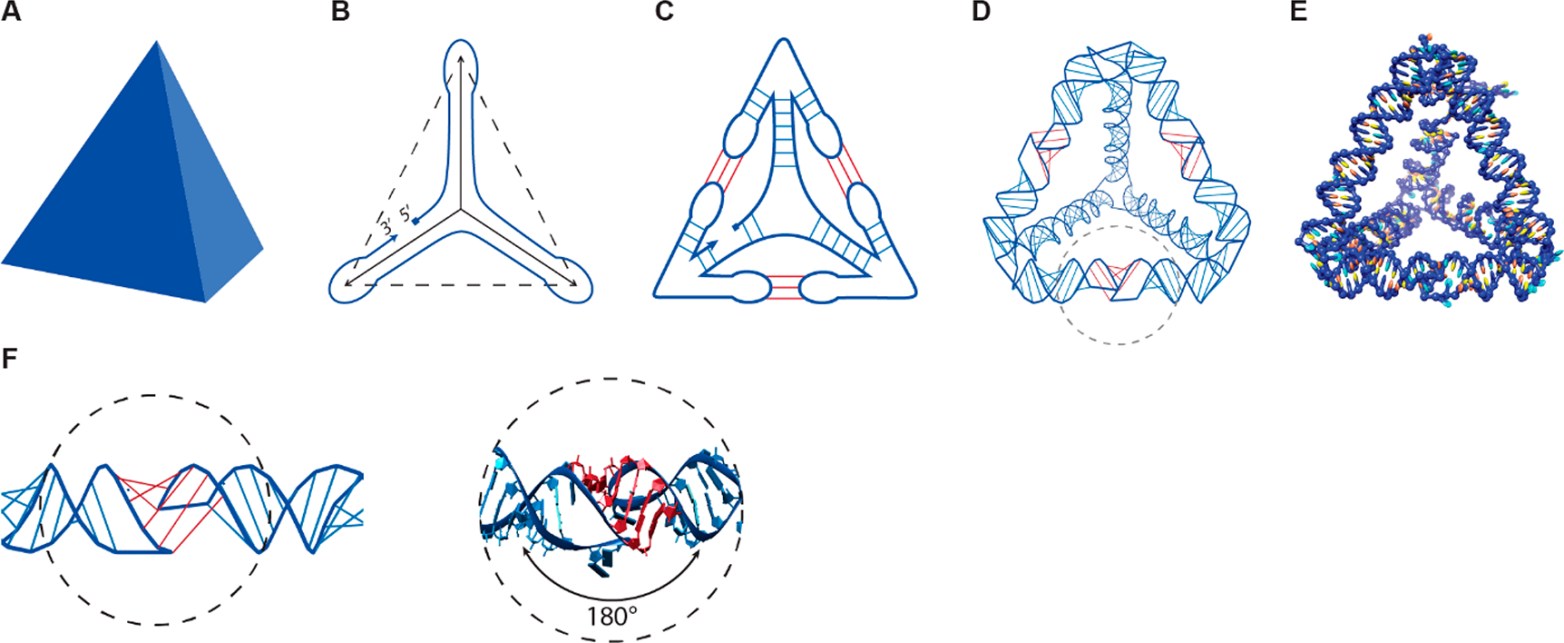
RNA polyhedron design scheme and HIV-DIS 180° kissing loop. (A) Targeted polyhedral model. (B) Spanning tree and strand routing of the polyhedral mesh. (C) Routing-based stem and kissing-loop pairings. (D) Helix-level model and (E) nucleotide-level model. (F) Schematic representation of the kissing loop and nucleotide-level model of the kissing-loop base pairing (Protein Data Bank ID: 1K9W).^29^

We describe our general design scheme using the simplest example, a tetrahedron (Figure 1A–E). The starting point is a polyhedral model (Figure 1A), whose wireframe representation we wish to render as a tertiary RNA structure (Figure 1E).

The first step is to create the RNA secondary structure of the targeted wireframe shape, deferring the precise nucleotide sequence design. We aim to render the edges of the wireframe mesh as RNA A-type helices, and toward that goal, we wish to route the RNA strand around the edges of the mesh in such a way that every edge is covered twice by the routing, in antiparallel directions. With an appropriate nucleotide sequence design, the complementary strand segments will then hybridize together to create the duplex edges.

However, complete antiparallel double routings of polyhedral meshes that keep all vertices intact exist only under quite special conditions,^30,31^ and in particular, for a tetrahedron such a routing is impossible. A way around this constraint is to first reduce the set of edges in the mesh to one of its spanning trees (a cycle-free set of edges that connects all the vertices),^30^ perform a strand routing on this tree, and then reintroduce the discarded edges using some connector motif. This design technique for single-stranded constructs goes back at least to Shih and co-worker’s mostly single-stranded DNA octahedron^4^ and has resurfaced many times, with the connection to spanning trees being made explicit by Veneziano et al.^13,28^ A schematic of the routing on a spanning tree of the tetrahedron mesh is presented in Figure 1B, where the three solid lines indicate the chosen spanning tree edges, the three dashed lines the discarded nonspanning tree edges, and the blue curve the routing of the strand around the spanning tree, with a nick between the 3' and 5' ends of the strand.

The connector we use to create the non-spanning tree edges is the 180° HIV-DIS kissing-loop motif successfully employed by Geary et al.^21^ This extrahelical pseudoknot pairing of two hairpin loops induces an almost perfect 180° alignment of the respective loop stems, and thus combines two separate “semi-helices” into an effectively contiguous helical structure (Figure 1F).

As outlined in Figure 1C, we extrude the strand routing at every vertex of the mesh by a half-edge hairpin loop along each discarded non-spanning tree edge and design the base sequence at the loop terminus to pair with the corresponding half-edge that protrudes from the other end-vertex of the edge. The kissing-loop pairing of the matching termini will then reintroduce the non-spanning tree edges to the structure.

A small number of unpaired nucleotides are added to each vertex of the structure to provide flexibility and thus facilitate the folding of the 3D conformation. The exact number of these is determined by an optimization process that also tries to match the rotation phases of the helices incident to each vertex, so that the cross-vertex transition of the RNA strand from one helix to the next creates minimal strain to the conformation. (For more details of the design process, see Supporting Information Text S1.)

Figure 1D presents a helix-level model of the resulting RNA polyhedron, with the regular intrahelical nucleotide pairings marked in blue and the extrahelical kissing-loop pairings in red.

A nontrivial aspect of the design process that is worth mentioning is the exclusion of strand routings that create mathematical knots and hence potential topological hindrances for successful folding. When discussing knottedness, we consider the strand to be a closed loop where the 3′-to-5′ nick is sealed. In practice, the RNA strand of course folds from an open conformation, so knotting is not an absolute obstacle, but nevertheless may lead to kinetic traps in the folding process. So, to ensure an unknotted routing, we imagine each vertex point as expanded into a small sphere and choose for each incoming strand segment an outgoing segment in such a way that the connecting “virtual” routings on the surface of the sphere do not cross each other. This property can always be achieved by a judicious geodetic point-matching protocol and can be proved by a simple topological argument to guarantee that the resulting global routing is an unknot. (For details, see Supporting Information Text S1.) This general way of treating the knotting problem is also what allows our design method to cover not just polyhedral but even arbitrary straight-line 3D meshes.

We have developed a software tool, *S*panning *T*ree *E*ngineered *RNA* design (*Sterna*), that automates the secondary-structure design process described above.^32^ This tool has been implemented as a Python add-on module to the open-source *Blender* 3D graphic design software suite.^33^ To initiate a design task, the user creates a model of the desired polyhedron using the tools in the *Blender* suite or imports an existing model from an external library. Pressing a “generate” button then performs the strand routing, creates the corresponding spatially embedded RNA A-helices, aligns their phases, and adds linker nucleotides at the vertices. The outcome can be viewed and edited on the Blender viewport screen or exported as a structured text file of type *snac* (*S*imple *N*ucleic *A*cid *C*ode), which contains a representation of the resulting secondary structure in the standard “dots-and-brackets” notation, together with 3D coordinates of the nucleotides along the helices.

The *snac* file can then be carried to a further module *snacseq*, which complements it with kissing-loop base sequences optimized for binding strength and mutual orthogonality, and a full primary structure sequence designed with the help of the NUPACK package.^34,35^ An additional module *snac2ox* can be used to transform this full *snac* file into input files for the *oxDNA* molecular dynamics simulation and visualization package.^36,37^ (For further information, see Supporting Information Note S1 and the software tutorial on the *Sterna* website.)^32^

**Figure 2:**
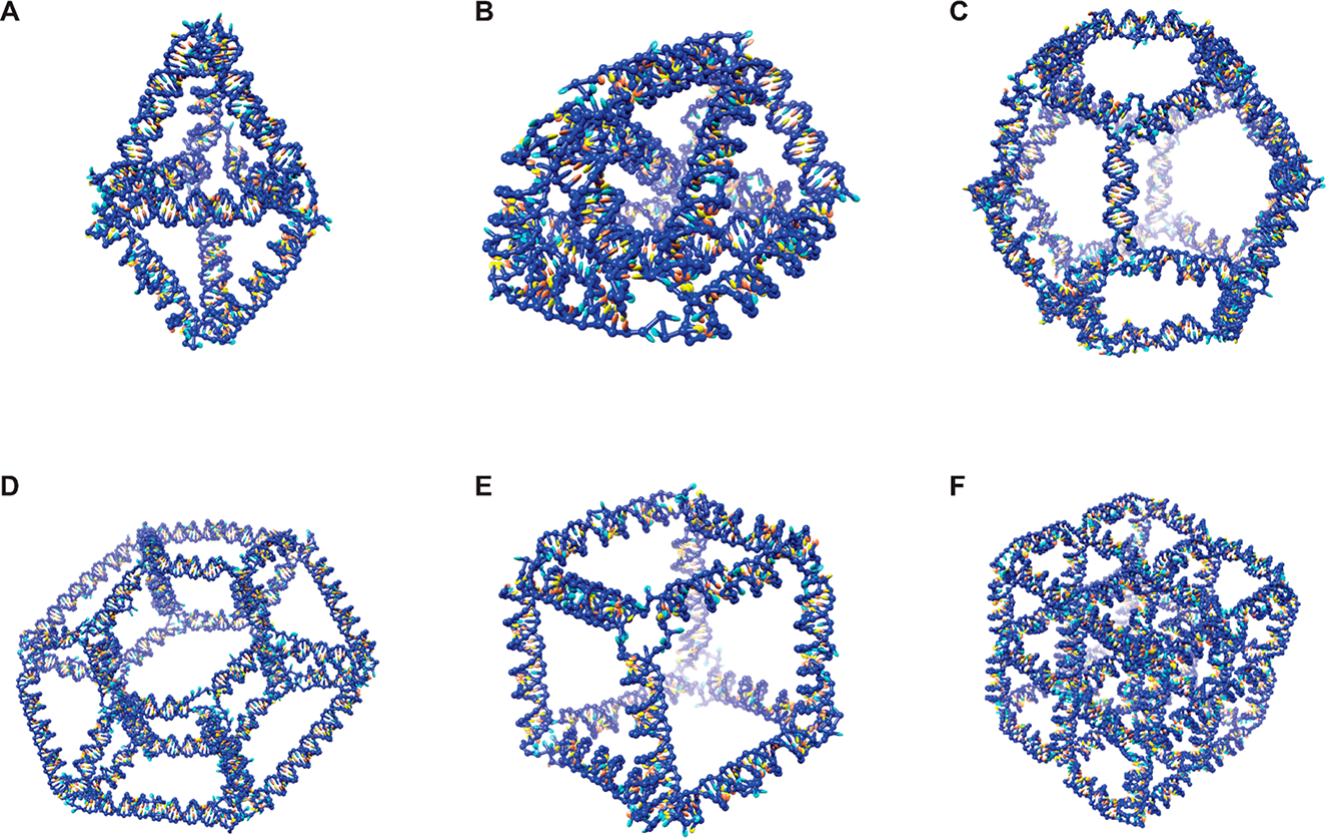
*oxDNA* models of *Sterna* designs for RNA meshes. (A) Bipyramid. (B) Prism (sides triangulated). (C) Dodecahedron. (D) Toroid. (E) Cube. (F) 2 × 2 × 2 Cube.

Figure 1E illustrates the complete nucleotide-level model generated by the *Sterna* secondary structure design tool and the *snacseq* primary-structure creation module from the *Blender* design of a tetrahedron shown in Figure 1A. The *snac2ox* module has been used to export the resulting design into *oxDNA*, which has then been used to relax the initial helix arrangement, and the output has been visualized using the *UCSF Chimera* package.^38^Figure 2 presents six further examples of *oxDNA/Chimera* renderings of *Sterna* designs for RNA meshes illustrated in the same way: Bipyramid (Figure 2A), Prism (Figure 2B), Dodecahedron (Figure 2C), Toroid (Figure 2D), Cube (Figure 2E), and 2 × 2 × 2 Cube (Figure 2F). Note that the 2 × 2 × 2 Cube mesh is not polyhedral, i.e., it does not derive from a polyhedral model and cannot be embedded on a 3D surface.

To validate our methodology, we synthesized three relatively small and distinctly characterizable 3D wireframe structures that were designed using the *Sterna* tool and the *snacseq* primary-structure generator: a Tetrahedron (Figure 1E), a Bipyramid (Figure 2A), and a Prism (Figure 2B). (Note that the sides of the Prism structure have been triangulated to ensure structural rigidity.) In addition, the aforementioned structures with some or all kissing loops replaced with nonpairing sequences were used for comparing their folding efficiencies. The secondary structures and sequences of all the designs are presented in Figure S7 and Table S3. In comparison to previous work, our Tetrahedron structure is somewhat smaller than the one designed by Li et al.,^25^ at 435 nt versus 623 nt, whereas our Bipyramid and Prism structures are larger, at 643 and 781 nt, respectively.

We did not try to synthesize the plain Cube (867 nt), because it was known theoretically and from simulations to be not rigid, and the Prism seemed like a more interesting target. The larger structures, Dodecahedron (1547 nt), Toroid (2191 nt), and 2 × 2 × 2 Cube (2569 nt) are included in Figure 2 only as illustrations of the design method and the capabilities of the *Sterna* tool.

**Figure 3:**
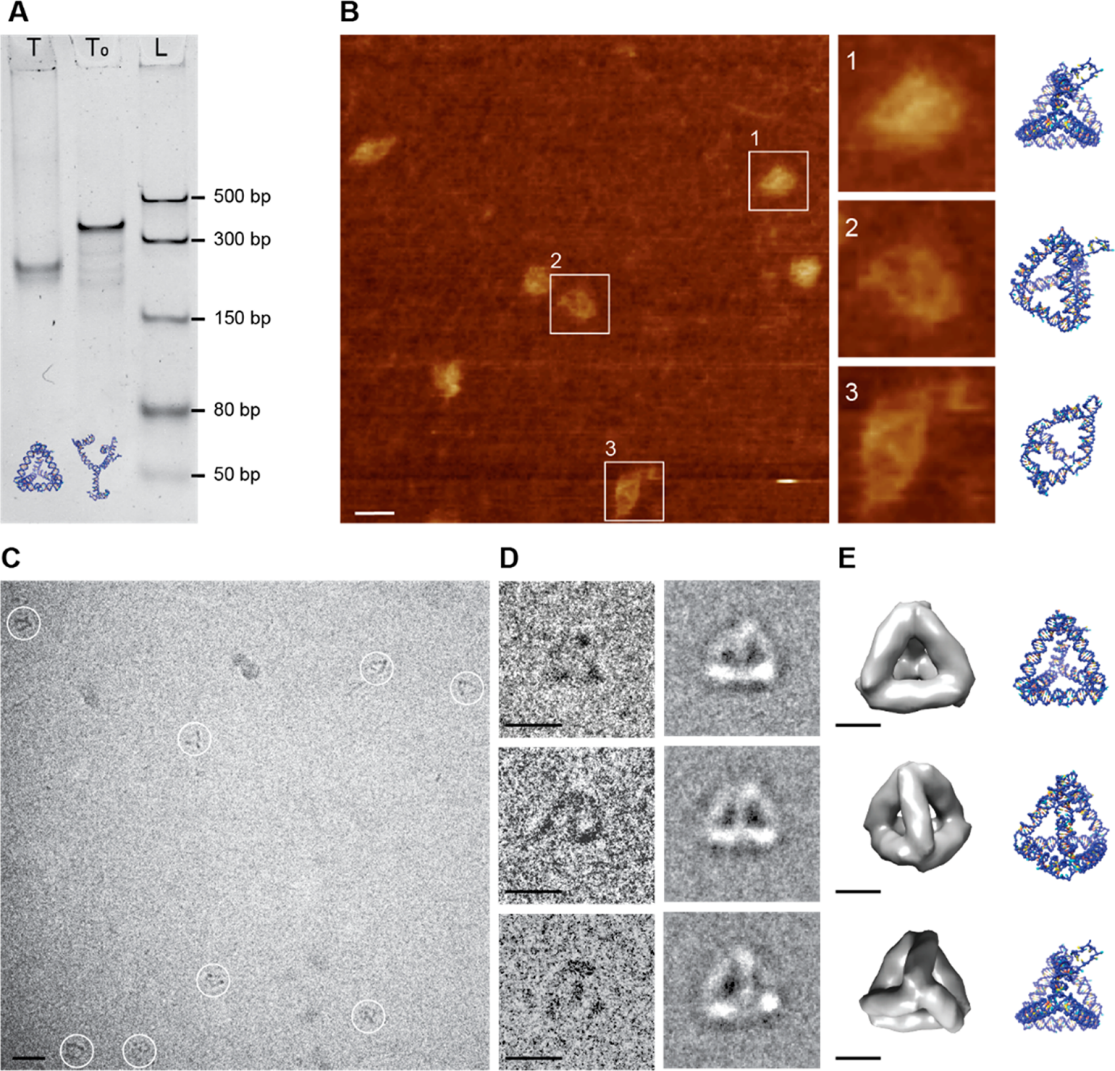
Characterization of Tetrahedron: (A) 5% native PAGE analysis of tetrahedra T and T_0_ folded at 50 nM concentration. A significant difference in migration speed can be observed between T and T_0_, suggesting that T is folded completely into a compact tetrahedron by the interaction of kissing loops. (B) AFM micrograph of Tetrahedron T. The squares marking the particles and the enlarged images (right) are 30 × 30 nm^2^ in dimension. Scale bar: 20 nm. (C) Cryo-EM micrograph of the Tetrahedron T sample. White circles indicate individual Tetrahedron particles; the circles have a diameter of 20 nm. The scale bar is 20 nm. (D) Individual particles picked from the micrographs (left) and their class averages (right). Scale bars: 10 nm. (E) Corresponding views of the Tetrahedron T structure reconstructed with 22 Å resolution from 1020 particles (left) and their *oxDNA* simulation models (right). Scale bars: 5 nm.

Tetrahedron (T) structures were chosen for the initial self-assembly for their simplicity compared to the other structures. The 435 nt long single-stranded RNA (ssRNA) for structure T was transcribed from the DNA template and purified using denaturing PAGE gel (poly acrylamide/bis-acrylamide). The purified ssRNA was folded by annealing in the folding buffer, resulting in the formation of hairpin duplexes and three kissing loops. The native PAGE results show that a distinct construct presumed to be T folded at concentrations of up to 100 nM (Figure S8A). There was also an additional slow-running band, which might be an aggregate of two or more T constructs. The tetrahedral structure T was compared with a deficient structure T_0_, where for each kissing loop, one of the hairpin-loop sequences was replaced with a tetraloop sequence, e.g., GAAA, and the other with a poly-AU sequence, e.g., AUAUAU. Hence, the kissing loops do not close in T_0_, and the structures do not fold into tetrahedral conformations. The native PAGE analysis shows that T_0_ migrate distinctly more slowly than T, suggesting that the observed T are indeed completely folded Tetrahedra, which are more compact than T_0_ (Figure 3A).

The construct T was characterized further using atomic force microscopy (AFM) and cryo-electron microscopy (cryo-EM). AFM imaging performed on T indicates that the structures were completely folded and correspond to geometric tetrahedra in various projections (Figure 3B). The enlarged AFM images 1 and 2 in Figure 3B witness the tetrahedral symmetry of T, corroborating the PAGE results. The enlarged AFM image 3 in Figure 3B shows a Tetrahedron structure with one broken kissing loop. We surmise that the AFM imaging itself causes the structures to open as the tip moves over the sample, which could explain the presence of partly unfolded structures observed in the image.

Further confirmation of correct folding was provided by cryo-EM analysis. The initial cryo-EM imaging of T folded at 100 nM yielded an extremely low density of structures (<1 particle per frame). The structures had an affinity toward the carbon in the grid, leaving very few structures in the hole at this concentration.

To increase the number of structures per frame, the sample was concentrated by spin filtering to ∼400 nM. The 4-fold increase in concentration reduced the time and effort in data collection as the density of structures increased significantly to >3 particles per frame (Figure 3C). In total, 1020 particles were picked, class-averaged, and reconstructed. The particles picked and their corresponding class averages show the different projections of construct T (Figure 3D). The reconstruction revealed the tetrahedral structure of T with a resolution of 22 Å, and the views corresponding to the class averages are presented in Figure 3E. The reconstruction performed without imposing any symmetry also showed a tetrahedral structure, confirming that upon annealing the ssRNA of T would fold into a Tetrahedron via formation of the kissing loops (Figure S9A, C1).

**Figure 4:**
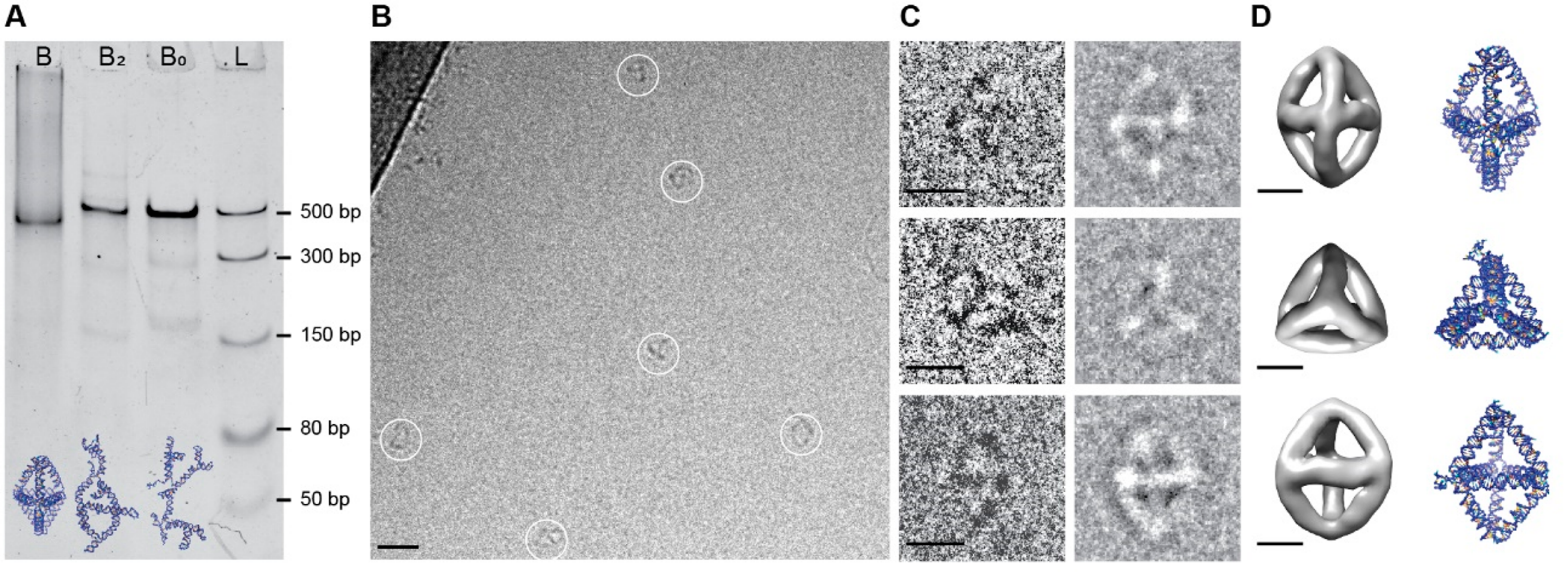
Characterization of Bipyramid: (A) 5% native PAGE analysis of Bipyramid B, partially deficient Bipyramid B_2_, and fully deficient Bipyramid B_0_. The faster running band at B indicates the folded Bipyramid. B_2_ and B_0_ run at similar speeds. (B) Cryo-EM micrograph of the Bipyramid B sample. White circles indicate individual Bipyramid particles; each circle has a diameter of 20 nm. Scale bar: 20 nm. (C) Individual particles picked from the micrographs (left) and their class averages (right). Scale bars: 10 nm. (D) Different views of the Bipyramid B structure reconstructed from 955 particles (left) and their corresponding *oxDNA* simulation models (right). Scale bars: 5 nm.

The Bipyramid (B) and Prism (P) structures were more complex, containing 5 and 7 kissing loops, respectively, compared to 3 in structure T, which made characterization of these structures more challenging. The native PAGE analysis of B folded at different concentrations shows only little aggregation (Figure S8B). In addition to B with five kissing loops, we also folded control structures B_2_ and B_0_, in which 3 or all 5 of these kissing loops were replaced by tetraloop and poly-AU sequences. Construct B ran faster than B_2_ and B_0_, indicating the fully folded nature of B (Figure 4A). The samples B_2_ and B_0_ migrated at similar speeds. This was somewhat unexpected, since B_2_ still had two kissing loops intact, compared to none in B_0_. To understand this migration behavior, we calculated the radius of gyration (*k*) value for all the structures from the results of *oxDNA* simulation runs using Visual Molecular Dynamics (VMD). The mean values of *k* for structures T, T_0_, B, B_2_, B_0_, P, and P_0_ were 5.33, 8.98, 6.29, 8.68, 9.27, 6.37, and 9.88 nm, respectively **(**Figure S10). We hypothesize that the close mean *k* values of B_2_ and B_0_ are the reason for their observed similar migration speeds.

The AFM imaging of construct B did not yield any significant information due to the complexity of the structure. Hence, the construct was imaged under cryo-EM at ∼400 nM concentration (concentrated using spin filtering from samples folded at 100 nM). The density of structures (∼2 particles/frame) was considerably lower than for the T structure (Figure 4B). Individual particles were picked from the cryo-EM micrographs and class averaged. The particles picked and their class averages illustrate the different projections of the Bipyramid (Figure 4C). The corresponding views of construct B after reconstruction using 955 particles with a resolution of 25 Å are presented in Figure 4D, along with their *oxDNA* simulation models. The reconstruction of the Bipyramid without imposing any symmetry presented in Figure S9B, C1, also confirms the folding of the ssRNA template into a Bipyramid.

The Prism (P) structure contains seven kissing loops and folded without any aggregates at 10 nM concentration; however, at concentrations ≥20 nM the sample started to aggregate, and only a small amount of correctly folded structures migrated to form a faint band (Figure S8C). We compared the folding capability of P with that of the other two structures folded at 50 nM concentration by running the corresponding samples in parallel in native PAGE (Figure S8D). The constructs T and B folded without any aggregation while P had aggregates stuck in the gel pocket. Also, there was a second band close to the pocket, which might be multimeric Prism structures formed by intermolecular kissing loop interactions. It could be observed that the migration speeds of T, B, and P are determined by both the radius of gyration and the length of the ssRNA strand. Though B and P do not have a significant difference in radius of gyration (Figure S10), they migrate at different speeds because of their different strand lengths of 643 and 781 bases, respectively.

**Figure 5:**
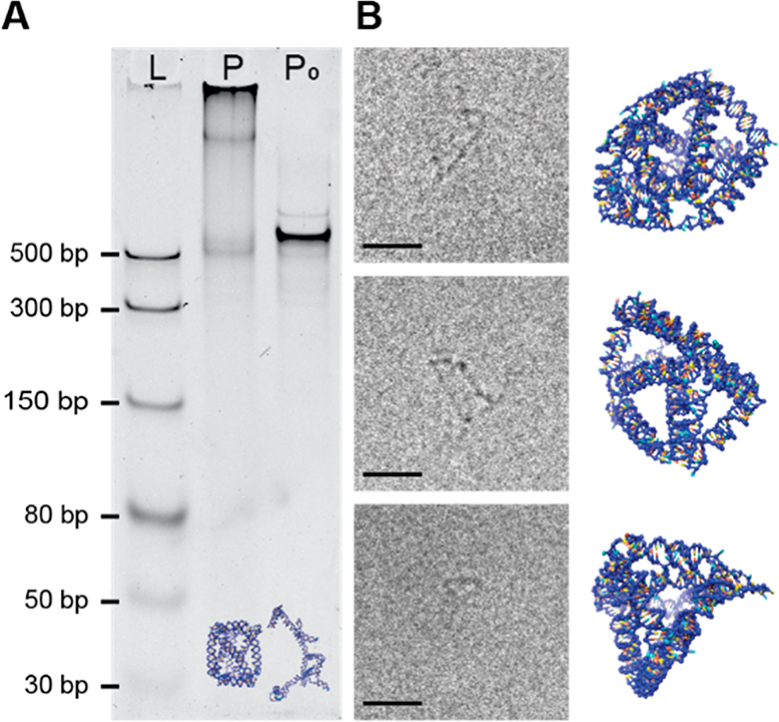
Characterization of Prism: (A) 5% native PAGE analysis of Prism P and P_0_. The Prism P structures exhibited severe aggregation and a slow-running band. The band running faster than P_0_ indicates the fully folded prism P. (B) Particles picked from cryo-EM micrographs (left) and their corresponding *oxDNA* simulation views (right). Scale bars: 20 nm.

For comparison, a structure P_0_ with all the kissing loops replaced with tetraloops and poly-AU sequences was synthesized. The sample P_0_ ran as a single strong band, which was slower than sample P, indicating the fully folded nature of the P particles (Figure 5A). We faced similar issues with AFM imaging of P as with B. The AFM could not resolve the structure and the images were noninformative. Though the prism structure appears to fold well at low concentrations (<20 nM) (Figure S8C), our efforts to concentrate the Prism P sample with spin filtering also did not result in high yields, making cryo-EM imaging time-consuming. A substantial portion of the structures found in the micrograph were either aggregated or had high affinity to the carbon in the grid. This resulted in fewer than 0.1 structures per frame on average. Some of the structures that were found intact and fully folded are presented in Figure 5B, along with corresponding projections from the simulation model, suggesting the targeted prism structure was indeed realized.

## Conclusions

We have presented a general high-level and fully automated design scheme for rendering 3D wireframe polyhedra as native conformations of single-stranded RNA molecules. An open-source distribution of the design software is available on the *Sterna* website.^32^ The method has been demonstrated by designing, synthesizing, and characterizing three small-to-moderate sized structures: a tetrahedron (435 bases), a triangular bipyramid (643 bases), and a triangular prism (781 bases). For the tetrahedron and the bipyramid, the yield of the synthesis was high, and we were able to obtain excellent cryo-EM reconstructions. In the case of the prism, a band of properly folded particles could be extracted, but the yield was too low to obtain decent quality reconstructions from the available number of cryo-EM grids. In further work, we will investigate ways of counteracting this effect, taking into account the recent experimental advances of Li et al.,^25^ Liu et al.,^26^ and Geary et al.^27^ For instance, we will investigate the impact on folding using stepwise annealing programs, different salt conditions and strand concentrations. We will also consider the effects of different spanning tree choices for a given mesh, the binding strength of kissing-loop pairs as affected by the length of the pairing domains, and the rigidity of mesh vertices as affected by the number of linker bases. Optimization of the design and experimental details, together with improvements in commercially available strand templates will allow us to pursue the synthesis of bigger structures: in principle, our design approach sets no limits on the size or complexity of the target meshes, although in practice, more attention will likely need to be devoted to RNA sequence design, e.g., to the design of good kissing-loop ensembles.

RNA nanostructures can be used as templates for functional molecules such as nucleic acids, small molecules, and proteins. For example, RNA nanostructures have been reported to image RNA–protein interactions, spatially organize proteins and enzymes, perform computation, target tumors and metastasis, deliver drugs, and control cellular functions such as gene expression and cell death.^15,39,40^ Recently, Liu et al.^41^ utilized the kissing-loop interactions in RNA origami to functionalize target RNAs and assemble them into closed homomeric nanoarchitectures for cryo-EM imaging of target RNA. Further developments in this direction should be explored to make RNA origami more robust and functional, similar to DNA origami.

## Methods

### Sequence Design

Sequences for the RNA nanostructures excluding kissing loops were designed using the design function of NUPACK.^34,35^ Six kissing-loop sequences used for the design were taken from existing literature,^42−44^ and the rest were manually designed six-base loops that are nonsimilar to others. (The *snacseq* tool provides a kissing-loop ensemble generator, but for the present experiments, proven models from the literature, complemented with manual designs, were used to decrease the number of possible sources of difficulty.)

A restriction was imposed on the sequences to prevent any of the following patterns: AAAA, CCCC, GGGG, UUUU, KKKKKK, MMMMMM, RRRRRR, SSSSSS, WWWWWW, YYYYYY. In the corresponding complementary sequences, GT mismatches were introduced at 8 base pair intervals to avoid creating a self-complementary sequence. The mismatches were necessary to synthesize the DNA templates as dsDNA in the form of custom gBlock gene fragments from Integrated DNA Technologies (IDT, Inc.).

All the sequences begin with the cap sequence GAC followed by the T7 promoter sequence TAATACGACTCACTATAG. The cap and promoter sequences are also used as a sequence for forward primer during PCR. The designs also have a 15 nt tail sequence in addition to the sequence required for the nanostructure. Different 15 nt tail sequences were used for the Tetrahedron, Bipyramid, and Prism, with the intention of using the 15 nt sequence as a reverse primer. However, to optimize the melting temperature (*T*_m_) of the forward and reverse primers, slightly longer reverse primer sequences were used. See Supporting Information Note S2 for the detailed script to design the DNA template sequence using NUPACK.

The DNA templates for all RNA nanostructures were ordered from IDT, Inc. as custom gBlock gene fragments. The primer sequences for PCR amplification were ordered from IDT, Inc. All the DNA sequences and the primers used can be found in Table S3.

### DNA Amplification and RNA Synthesis

The gBlocks from IDT were resuspended in nuclease-free water, and the final concentration was 10 μg/μL. The DNA templates were amplified over 20 cycles using Phusion High-Fidelity DNA Polymerase (New England Biolabs, Inc.). The final concentration of components in a 100 μL PCR reaction was 1 ng/μL of DNA template, 0.5 μM of forward and reverse primers and 1× Phusion High-Fidelity PCR Master Mix with HF Buffer. The reaction mixture was denatured initially at 98 °C for 30 s, followed by cycles of 8 s at 98 °C, 15 s at 58 °C, and 30 s at 72 °C. The final elongation step was for 7 min at 72 °C. The PCR product was purified using Monarch PCR & DNA Cleanup Kit (New England Biolabs, Inc.). The samples were run on a 1.5% Agarose (Merck) gel with SyBr Safe (ThermoFisher Scientific) in 0.5× TBE buffer at room temperature for 60 min at 120 V. The gels were imaged using Bio-Rad Gel Doc XR system. The purified PCR DNA templates (∼20 ng/μL) were transcribed in a reaction mixture containing 4 mM of each rNTP, 12.5 mM of MgCl_2_, 1× RNA polymerase buffer, and ∼1 U/μL of T7 RNA polymerase (New England Biolabs, Inc.). The samples were incubated at 37 °C for 6 h, and an additional hour after the addition of 2 U/50 μL of DNase (New England Biolabs, Inc.). The RNA samples were purified using an 8% poly acrylamide/bis-acrylamide (29:1) (PAGE) gel containing 8 M urea and 1× TBE. The samples were preheated at 95 °C for 5 min and loaded on the gel. The samples were run at 58 °C for 100 min at 100 V. The gel was post-electrophoresis stained with SyBr Green (ThermoFisher Scientific) for 15 min and imaged using Bio-Rad Gel Doc XR system. The bands of interest were cut from the denaturing gels and purified using ZR small-RNA PAGE Recovery Kit (Zymo Research). The purified RNA samples were stored in nuclease-free water at −20 °C.

### Assembly of RNA Nanostructures and Characterization

The RNA samples were thermally annealed in a folding buffer containing 0.5× TE buffer with 1 mM MgCl_2_ and 100 mM NaCl. For each nanostructure, 100 nM of ssRNA in folding buffer was rapidly folded by heating to 80 °C for 5 min followed by cooling to 20 °C at 0.1 °C/s. The samples were run in a 5% native PAGE gel in an ice bath along with a low-range ssRNA ladder (New England Biolabs, Inc.). The gels were prepared using acrylamide/bis-acrylamide (29:1) containing 1 mM MgCl_2_ and 100 mM NaCl. The gels were post-electrophoresis stained with SyBr Green (ThermoFisher Scientific) for 15 min and imaged using Bio-Rad Gel Doc XR system.

### AFM Imaging

Atomic force microscopy (AFM) imaging was performed using a tip-scan high-speed AFM (BIXAM, Olympus, Tokyo, Japan) that was improved based on a previously developed prototype AFM.^45,46^ A freshly cleaved mica surface was pretreated with 0.05% 3-aminopropyltriethoxysilane (APTES).^47^ A drop (2 μL) of the sample (about 1 nM) in the TAE-Mg buffer (40 mM Tris-acetate, 1 mM EDTA, 2 mM MgCl_2_, pH 8.3) was deposited onto the APTES-treated mica surface and incubated for 3 min. The surface was subsequently rinsed with 10 μL of the TAE-Mg buffer. Small cantilevers (9 μm long, 2 μm wide, and 100 nm thick; USC-F0.8-k0.1-T12, NanoWorld, Switzerland), with a spring constant of ∼0.1 N/m and a resonant frequency of ∼300–600 kHz in water, were used to scan the sample surface. The 320 × 240 pixel^2^ images were collected at a scan rate of 0.2 frames per second (fps). The imaged sequences were analyzed using AFM scanning software (Olympus) and ImageJ (http://imagej.nih.gov/ij/) software.

### Cryo-EM Imaging and Single-Particle Reconstruction

For cryo-EM, 100 nM RNA polyhedra samples folded in TE/Mg^2+^/Na^+^ buffer were concentrated using 3 kDa MWCO Amicon Ultra centrifugal filters (Merck) to a final concentration of ∼400 nM. Next, 5 μL of the concentrated sample was applied on 300 mesh Cu grids coated with lacey carbon (Agar Scientific). The grids were blotted for 3 s (70% relative humidity) by Leica EM GP2 plunge freezer followed by immediate vitrification using liquid ethane (−170 °C). Vitrified samples were cryo-transferred to the microscope and imaged using a JEOL JEM-3200FSC TEM while maintaining specimen temperature of −190 °C. EMAN2^48^ was used for single-particle reconstruction of the RNA nanostructures. The reconstruction of the Tetrahedron was performed with 1020 particles that were used for reference-free class averaging. The initial models were generated by imposing cyclic C1 (no symmetry) or tetrahedral symmetry (TET) and refined with C1, C3, and TET symmetry. For the Bipyramid reconstruction, 955 particles were picked for class averaging, initial reconstruction, and refinement. The reconstructed models were visualized using *UCSF Chimera*.

## Supporting Information Available

Design of polyhedral structure and sequences, primer and polyhedral model DNA sequences, and other supporting data (PDF)
Movies of short *oxDNA* simulation traces visualized as *Chimera* movies (MP4, MP4, MP4)
